# Selective Adsorption of Potassium in Seawater by CoHCF Thin Film Electrode and Its Electrochemical Desorption/Regeneration

**DOI:** 10.3390/ma14133592

**Published:** 2021-06-27

**Authors:** Nan Zhang, Tohru Kawamoto, Hiroshi Watanabe, Yong Jiang, Zhenya Zhang, Zhongfang Lei, Durga Parajuli

**Affiliations:** 1 Nanomaterials Research Institute, National Institute of Advanced Industrial Science and Technology (AIST), 1-1-1 Higashi, Tsukuba 305-8565, Japan; zhangnan01@caas.cn (N.Z.); tohru.kawamoto@aist.go.jp (T.K.); watanabe-hiroshi-hia@aist.go.jp (H.W.); jysilicon@hotmail.com (Y.J.); 2 Graduate School of Life and Environmental Sciences, University of Tsukuba, 1-1-1 Tennodai, Tsukuba 305-8572, Japan; zhang.zhenya.fu@u.tsukuba.ac.jp (Z.Z.); lei.zhongfang.gu@u.tsukuba.ac.jp (Z.L.)

**Keywords:** CoHCF thin film, seawater, potassium recovery, electrochemical desorption, intraparticle diffuse model

## Abstract

Cobalt Hexacyanoferrate (CoHCF) was tested for the selective uptake of K from seawater and the electrochemical method was adopted for the desorption and regeneration of the material. Powder form CoHCF could adsorb about 6.5 mmol/g of K from the seawater. For the ease of the electrochemical desorption and regeneration, CoHCF thin film was coated onto the Indium Tin Oxide (ITO) glass to obtain a CoHCF electrode. K adsorption kinetics on CoHCF thin film was found to be well fitted with the intraparticle diffusion model, which was a two-step process. Five consecutive adsorption-desorption-regeneration cycles were carried out to know the gradual decrease in the adsorption capacity owing to changes in the redox states of two metals, Co and Fe, in the material. Fourier Transform Infrared Spectroscopy (FT-IR) and Ultraviolet-Visible (UV-Vis) measurement results corresponded to the color change of CoHCF thin film, indicating the valence change of transition metals and the exchange of alkali metal cations happened on the CoHCF at different operation stages. In order to elucidate the reaction mechanism, composition of the material was analysis in the following steps: adsorption, desorption, and regeneration. It was proved that the system based on CoHCF thin film modified electrode had the potential of recovering potassium from seawater.

## 1. Introduction

Potassium is a crucial fertilizer component, the demand of which is ever increasing for reaching the food supply [1,2,3]. Together with potash ore mining, secondary sources such as urine, sewage water, and biomass leachate are also gradually being used as sustainable potassium resources [4,5,6,7]. Another reliable source of this valuable element can be the brine wasted from the seawater desalination system, which uses a rather new technology—reverse osmosis [8,9]. By using the brine, not only the increase in salinity of the drained area could be controlled [10], but also, the huge element resources can be used [11]. As a result, along with the environmental sustainability, some economic value can be expected [12]. Potassium is the fourth most abundant element in seawater. With 0.399 g/L concentration, it is next to Na^+^, Ca^2+^, and Mg^2+^. Sea brine contains about 2 g/L potassium [13].

Efforts are being made for the recovery of K^+^ from seawater. The traditional evaporation and membrane separation methods have shown some success with limitations [14,15,16]. The environmental factors, such as weather dependency and the use of a large area of land are the limiting factors of the evaporation-precipitation method [17,18]. In the precipitation process of K^+^ separation, anions such as dipicrylamine anion (DPA^−^) or pentaborate (B_10_O_16_^2−^) were used, which added to the toxicity problem [19,20].

A more feasible and advantageous method would be the selective adsorption of K^+^ from seawater [21]. Yet, the required selectivity factor would be case specific. In some aqueous matrices such as urine, the concentration of K^+^ (86.5 mmol/L) is similar to that of coexisting cations NH_4_^+^ (39.7 mmol/L) and Na^+^ (157 mmol/L) [22]. In the case of seawater, however, K^+^ concentration is much lower compared to the most abundant Na^+^. This means, for the successful recovery of potassium from the seawater, the first and foremost requirement is that the material must possess incomparable selectivity for K^+^ over Na^+^. Zeolites and ocean manganese nodules have already been studied for K^+^ removal from seawater [13,23,24]. Yet, these adsorbents have poor K^+^ capture ability due to the lack of specific selectivity. In this regard, Metal hexacyanoferrates (MHCF) can be highly likely materials.

MHCF is a class of coordination compounds with a generalized molecular formula of A_x_M[Fe(CN)_6_]_y_∙nH_2_O, where A is the alkaline cations of Na^+^ or K^+^, M stands for the transition metals Co, Ni, Cu, Fe, and so on [25]. As a pigment widely used from the 18th century, application of MHCF has been extended to Cs decontamination or the making of electrochromic device and recently for the recycling of ammonia and ammonium [26,27]. According to previous studies, MHCF highly selectively adsorb Cs^+^ and next comes Rb^+^. The positive aspect is that the concentration of these two elements in seawater is much lower than that of K^+^. In our recent study, CoHCF was found to have a good adsorption ability towards K^+^. For consideration of competing ions existing in seawater (Na^+^, Mg^2+^, Ca^2+^ etc), the selective adsorption capacity of K^+^ by MHCF should be carefully explored. Concerning the recovery of K^+^ after using the adsorption method, the desorption of K^+^ and the regeneration of the adsorbent is also of significance [28,29]. It is well known that the MHCF can be electrochemically reduced or oxidized by switching the applied potential. This phenomenon can be of great use for the adsorption, desorption, and regeneration of the material, generally called the electrochemically switched ion exchange (ESIX) process [30,31]. Based on this property, MHCF was applied as the electrodes of supercapacitors or K^+^ sensors [32,33]. Selective removal and recovery of cesium from seawater have already been achieved using CuHCF modified electrode in an electrochemical system [34].

This study is about the preparation of CoHCF-modified electrode and testing its ability for the selective adsorption of K^+^ from seawater [35]. K^+^ desorption and regeneration were carried out with the electrochemical method. The adsorption was studied taking simulated seawater. Dilute KCl solution was used as the electrolyte in the electrochemical desorption system aiming at the one step recovery of high purity potassium salt. The results demonstrate that the selective recovery of K^+^ from the seawater can be realized using the highly efficient MHCF modified electrodes.

## 2. Materials and Methods

### 2.1. Preparation of Powder-Form MHCFs 

For comparing the effect of different transition metals, Co, Cu, Ni, and Fe were used as for the M^II^ sites. MHCF synthesis was carried out by mixing sodium hexacyanoferrate (NaHCF) and MCl_2_ (M = Co^II^, Cu^II^, Ni^II^, Fe^II^) solutions, according to Equation (1):

(1) $$0.67{\mathrm{Na}}_{4}\left[\mathrm{Fe}{\left(\mathrm{CN}\right)}_{6}\right]+{\mathrm{MCl}}_{2}\to {\mathrm{Na}}_{0.68}M{\left[\mathrm{Fe}{\left(\mathrm{CN}\right)}_{6}\right]}_{0.67}+2\mathrm{NaCl}.$$

The synthesis of four kinds of MHCFs were carried out using the batch method: MCl_2_ in H_2_O (0.4 mol/mL, 10 mL) and Na_4_[Fe(CN)_6_] in H_2_O (0.268 mol/L, 10 mL) were mixed and shaken with 1000 rpm for 3 min at room temperature using a shaking incubator (SI-300C, AS ONE, Tokyo, Japan). 

For comparing the effect of different composition of CoHCF on K^+^ adsorption, CoHCF was synthesized by an assembled T-shaped micromixer with a 0.5 mm internal diameter. The preparation method of CoHCF with different R_mix_ (R_mix_ denotes the amount ratio of Na_4_[Fe(CN)_6_] to CoCl_2_ used in the synthesis) was done using the flow method, as described in our previous publication [35,36].

After the synthesis of the MHCFs in both batch and flow methods, washing with centrifugation at 12,000 rpm was done 3 times. Finally, MHCFs were obtained by vacuum drying at room temperature for 72 h.

### 2.2. Preparation of CoHCF Thin Film

The CoHCF slurry was prepared with the flow method using a micromixer in order to get particles with close size [37]. CoCl_2_·6H_2_O solution (0.6 mol/L, 50 mL) and Na_4_[Fe(CN)_6_]·10H_2_O (0.4 mol/L, 50 mL) solutions were prepared with pure water and passed through the micromixer (internal diameter: 0.15 mm) with a flow rate of 20 mL/min for each solution. After the CoHCF slurry was obtained, it was washed with pure water five times. The slurry was diluted with pure water and was filtered through a 0.45 μm membrane (Kiriyama Co. Ltd., Tokyo, Japan). The composition of the obtained CoHCF was assumed to be Na_0.67_Co[Fe(CN)_6_]_0.67_ (H_2_O was ignored). For making the NaCoHCF ink, 1000 mg poly(vinyl alcohol) solution (10 wt%) was added to 5000 mg CoHCF slurry and stirred overnight. The weight percentage of CoHCF ink was measured to be 7.03 wt%.

For the preparation of CoHCF thin film, ITO glass (2.5 cm × 2.5 cm) was first cleaned with active plasma and then used as the substrate. Two hundred μL of the above-mentioned ink was carefully dropped and spread over the ITO substrate. The spin coating process was carried out with a spin coater (ACT-300A, Active Spincoater, Kanagawa, Japan) at the flow of dry air with a two-stage coating method: 1200 rpm for 10 s and 1500 rpm for 20 s. At last, the ITO glass coated with CoHCF thin film was dried at 120 °C for 1 h.

### 2.3. K^+^ Adsorption from Powder-Form MHCFs

Batch adsorption experiments using powder-form adsorbents were carried out to show the effect of metal substitution and material composition. Artificial seawater from Yashima Pure Chemical Co., LTD, Osaka, Japan, was prepared for adsorption tests. The concentration of the main components in the original artificial seawater was listed in Table 1. In each batch adsorption experiment, 10 mg adsorbent was put in 10 mL of KCl-added artificial seawater and shaken in the thermostat shaker (SI-300C, AS ONE Corp., Tokyo, Japan) at 600 rpm under 30 °C for 22 h. The K concentration in seawater before and after adsorption was measured with NEXION 300D Inductively Coupled Plasma Mass Spectrometry (ICP-MS) (Perkin Elmer, Waltham, MA, USA) in the Kinetic Energy Discrimination (KED) mode. The adsorption experiment was repeated three times for each adsorbent and the standard deviation was obtained.

### 2.4. Adsorption Kinetics and Cycle Experiments for K^+^ Recovery

**Scheme 1:**
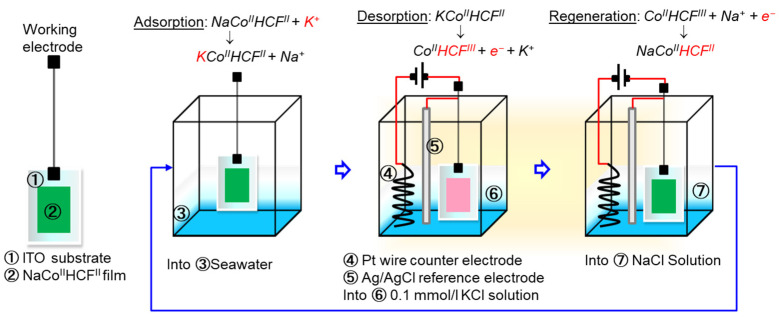
Process showing the adsorption of K^+^ onto NaCoHCF followed by its electrochemical desorption and regeneration.

As summarized in Scheme 1, the kinetic study and cycle experiments, adsorption tests were carried out in batch form with a NaCoHCF thin-film coated electrode (adsorbent loading amount: 1 mg) in seawater. In a typical trial, the NaCoHCF-electrode was put in a 50 mL sample tube containing 10 mL of artificial seawater under stirring using a magnetic stirrer for adsorption of 20 min. Adsorption kinetics were studied within a time span between 0 and 24 h. 

For the electrochemical desorption and regeneration in the cycle experiments, the CoHCF-electrode was set into a quartz cell with the inside size of 4 cm × 4.5 cm × 1 cm. For the counter electrode and the reference electrode, 10 mL 0.1 mmol/L KCl solution 2.5 m long Pt wire (φ 0.3 mm) and Ag/AgCl (saturated KCl) electrode, respectively, were used. A salt bridge conditioned with 0.1 mmol/L 1 NaCl solution to avoid the influence of K^+^ released from the reference electrode was used during the reaction. For K^+^ desorption, the potential of +1.0 V was applied for 1000 s. After the desorption, K^+^ concentration change in the electrolyte was investigated using ICP-MS. After the desorption, the CoHCF-electrode was put into 10 mL pure water for 3 min for removing the surface K^+^. The potential of −0.2 V was applied for 1000 s for the regeneration of the CoHCF-electrode back to the Na-containing form, using 10 mL 10 mmol/L NaCl as the electrolyte. Another cycle was tested for CoHCF-electrode after dipping in 10 mL pure water for 3 min. The adsorption-desorption-regeneration tests were repeated for 5 cycles.

### 2.5. Characterization of the NaCoHCF Thin Film

The composition of NaCoHCF was characterized by first dissolving the material using a Perkin Elmer-Anton Par Multiwave 3000 microwave decomposition system (Perkin Elmer, Waltham, MA, USA) followed by analyzing the Fe, Co, Na, K content using ICP-MS. Fourier Transmittance Infrared Spectrum was taken using a Thermo Fisher model Nicolet iS5 in ATR mode. UV-Vis spectrum was measured with a UV-Vis light source (DH-2000, Ocean Optics Inc, Dunedin, FL, USA) and data collection unit (USB4000, Ocean Optics Inc). The crystal size and structure were characterized by an X-ray diffractometry with Cu Kα at the wavelength of 1.5418 Å (D8 ADVANCE, Bruker AXS Inc., Billerica, MA, USA). Electrochemical measurement including cyclic voltammetry (CV) and chronocoulometric measurement was carried out using an electrochemical analyzer (CHI6115D, ALS/CH instruments, TX, USA).

## 3. Results and Discussion

### 3.1. K^+^ ion Adsorption Using Powder CoHCF

**Figure 1:**
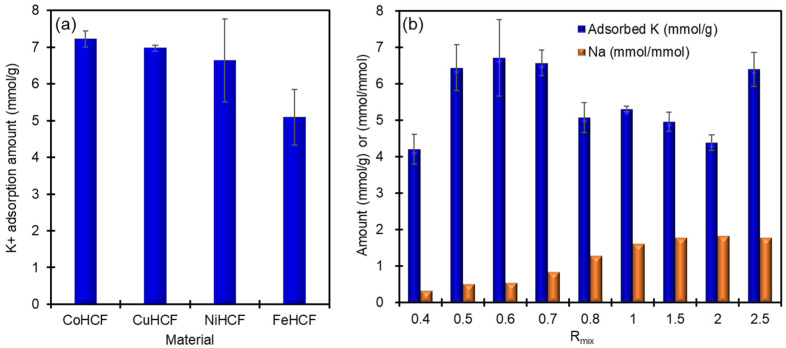
Potassium adsorption from KCl-added artificial seawater using different powder-form MHCF adsorbents. (**a**) MHCF with different transition metals (M = Co, Cu, Ni, Fe), and (**b**) K adsorption onto CoHCF prepared with different composition, R_mix_ from 0.4 to 2.5. 10 mg adsorbent, 10 mL solution, 600 rpm mixing for 24 h at 25 °C. Na (mmol/mmol) is the amount in the material. Because the change was negligible compared to the initial concentration, the amount of Na released was not analyzed.

Comparison of K^+^ adsorption by CoHCF, CuHCF, NiHCF, and FeHCF was shown in Figure 1a. CoHCF has a relatively higher K^+^ adsorption amount compared with the Cu, Ni, and Fe counterparts, showing the superiority of choosing Co as the transition metal of MHCF for K^+^ adsorption. For the various CoHCF synthesized at different R_mix_ (Figure 1b), K^+^ adsorption tests showed that the highest adsorption was acquired at a range of R_mix_ between 0.5– and 0.7. The tendency of composition influence on K^+^ adsorption capacity was slightly different than our previous results: K^+^ adsorption amount by CoHCF will increase with the exchangeable sodium ion number in CoHCF or the R_mix_ of preparation [35]. Higher R_mix_ yields the material with less vacancy, which means the cation exchange may not be as smooth. It can be seen that the actual K^+^ adsorption amount in seawater (up to 6.5 mmol/g) was higher than that of the theoretical value according to the cation exchange mechanism (~2.9 mmol/g). This excellent K^+^ adsorption ability of CoHCF can be attributed to another mechanism where a part of K^+^ is adsorbed into CoHCF lattice together with Cl^−^ anion [38]. This can explain the superior K^+^ adsorption ability in the given artificial seawater system with a large quantity of Cl^−^ concentration. From the K^+^ adsorption results, the suitable transition metal and condition of preparing the MHCF adsorbent can be determined. Finally, we decided to use CoHCF with R_mix_ of 0.67 for the preparation of CoHCF thin film and recovering K^+^ from seawater.

### 3.2. Characterization of CoHCF Thin Film

**Figure 2:**
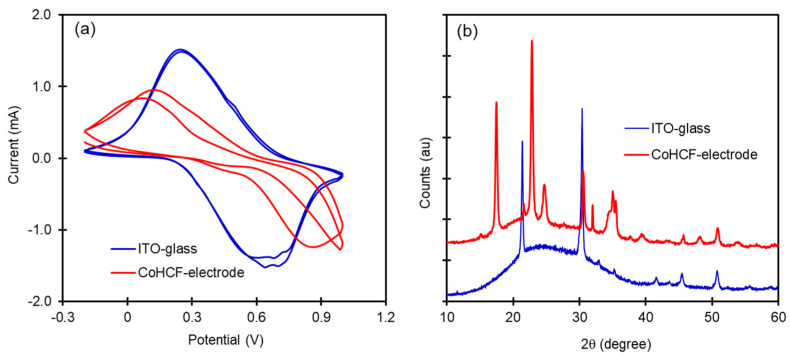
Characterization of CoHCF thin film. (**a**) Cyclic voltammograms of raw ITO glass and that coated with CoHCF thin film vs. Ag/AgCl electrode. Scan rate = 5 mV/s. (**b**) XRD pattern of CoHCF thin film on ITO glass.

CV curves of the fresh CoHCF thin film and raw ITO glass were taken and shown in Figure 2a. Compared to raw ITO glass, the current was increased after CoHCF modification, which means the modification of CoHCF thin film enhanced the electron migration and exchange between electrolyte and film. Redox peaks clearly appeared at 0.26 V/0.67 V, indicating the electrochemical redox reaction of CoHCF between this potential window, in accordance with the transition between the reduced and oxidized state of CoHCF with potential change.

From the XRD pattern shown Figure 2b, the characteristic peaks of CoHCF and ITO glass were identified. Through calculation using the Scherrer equation, Table 2, the average particle size of CoHCF in the thin film was obtained to be 178.4 nm, which is larger than its powder form reported in our previous study considering the aggregation during the ink preparation process [35].

### 3.3. K^+^ Recovery from Seawater by CoHCF Thin Film

**Figure 3:**
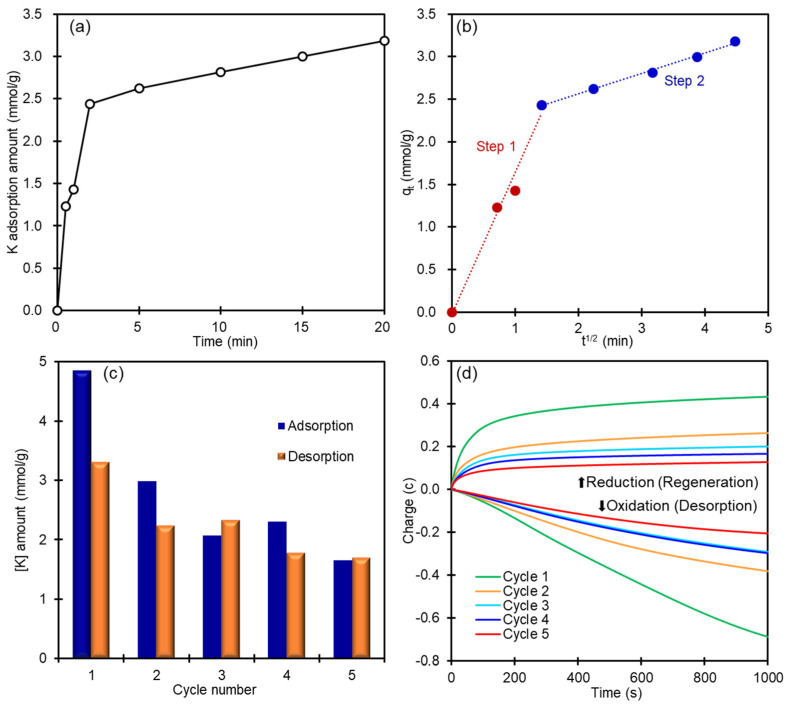
Potassium adsorption from artificial seawater using CoHCF modified electrode. (**a**,**b**) adsorption kinetic curve and intraparticle diffusion model, (**c**) Change of K^+^ amount in 5 adsorption-desorption-regeneration cycles, and (**d**) charge generated in each cycle of K^+^ adsorption and desorption in an electrochemical system.

From Figure 3a, it can be seen that the K^+^ adsorption amount increased with contact time and reached equilibrium after 20 min. The curve exhibited a tendency of quick adsorption in the first 2 min and then slowly turned to equilibrium. In the artificial seawater with K^+^ level to be around 400 ppm, the K^+^ uptake by CoHCF thin film was beyond 3 mmol/g after reaching equilibrium, which is higher than some previously reported adsorbents. Pan et al. reported an ocean manganese nodule as the adsorbent of potassium from seawater and the K^+^ adsorption capacity was 0.57 mmol/g [13]. Lei et al. studied a synergic removal of potassium and nitrate with kaolinite and Mg-Al layered double hydroxide; the adsorption ability for potassium was around 0.16 mmol/g [39].

The fractal-like property of K^+^ adsorption kinetics by CoHCF inspired the usage of the intra-particle diffusion model for the description of the adsorption kinetic curve [40]. The intra-particle diffusion model follows Equation (2) [41]:

(2) $$q_{t}=k_{i}\cdot t^{0.5},$$

where k_i_ represents the intra-particle diffusion rate constant (mg/g·min^1/2^), t stands for the time period from the beginning of the adsorption, q_t_ (mmol/g) means the adsorption quantity at time t. The intraparticle diffusion model can well describe the adsorption of K^+^ onto CoHCF thin film, which was found to be a two-step process. Adsorption was fast in the first 3 min and slowed down in the second step. The fitted straight line passed through the origin in the first step, reflecting that intraparticle diffusion was the rate-determining step in the first stage. In the second stage, the straight line deviated from the origin, reflecting that other mechanisms may also be responsible for the rate-controlling of K^+^ adsorption, such as cation exchange mechanism [42].

**Figure 4:**
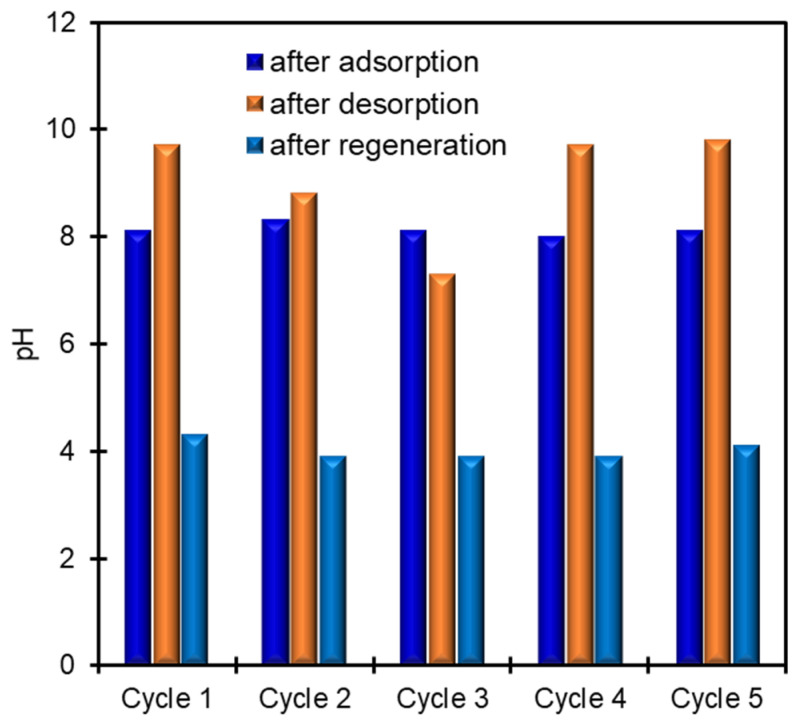
pH change at each cycle of adsorption-desorption-regeneration.

Cycle experiment was performed for five cycles to realize the recovery of K^+^ and recyclable usage of CoHCF thin film with electrochemical desorption and regeneration. Figure 3c exhibited the adsorption/desorption amount in five cycles and Figure 3d showed the charge produced in electrochemical processes. It was demonstrated that CoHCF had an adsorption ability of 4.84 mmol/g for K^+^ from seawater in the first cycle. In the first desorption process, 68.04% of the adsorbed K^+^ was released into the electrolyte. K^+^ concentration in the solution raised from 3.60 mg/L to 13.90 mg/L after the positive potential was applied. After the first cycle, the K^+^ adsorption amount dropped to 2.99 mmol/g in the second cycle and slowly decreased in the following cycles. There was also a tendency that the desorption ratio was kept above 74% since the second cycle, suggesting the electrochemical activation of CoHCF thin film after the first cycle. As shown in Figure 3d, the charge-t curves for desorption and regeneration were exhibited symmetrically at both sides of the x-axis. In order to evaluate the efficiency of the electrochemical reaction system on the material oxidation or cation releasing, Faraday’s efficiency in the desorption process was calculated, Table 3, and a relatively high value ranging between 37.07% to 63.84% was obtained. pH variation was found after the desorption/regeneration process (Figure 4), which should be attributed to the redox reaction of H_2_O on the counter electrode. The charge in the desorption/regeneration process gradually decreased from the first to the fifth cycle and could possibly be caused due to two reasons: first, the incomplete oxidation of Co(II)-Fe(II) in CoHCF or releasing of K^+^ cations; second, a small part of CoHCF thin film peeled from electrode substrate during reactions.

Faraday efficiency (FE) is calculated according to Equation (3):

(3) $$\mathrm{FE}\left(\%\right)=\frac{Q_{\mathrm{cal}}}{Q_{\mathrm{meas}}}=\frac{n\;\times \;F\;\times \;\left(c_{0\;}-{\;c}_{t}\right)\;\times \;V\;\times \;10^{-6}}{Q_{\mathrm{meas}}}\;\times \;100\%,$$

where Q_meas_ (C) is the generated charge amount according to charge-T graph; Q_cal_ (C) stands for the charge amount calculated from the equivalent amount of oxidation reaction; *n* stands for the number of electrons transferred during electrochemical reaction; *F* is the Faraday constant 96,485.33 C/mol; c_0_ and c_t_ (µmol/L) represent the K^+^ concentration before and after electrochemical reaction; and V (L) is the solution volume.

### 3.4. Spectroelectrochemical Characterization of CoHCF Thin Film

**Figure 5:**
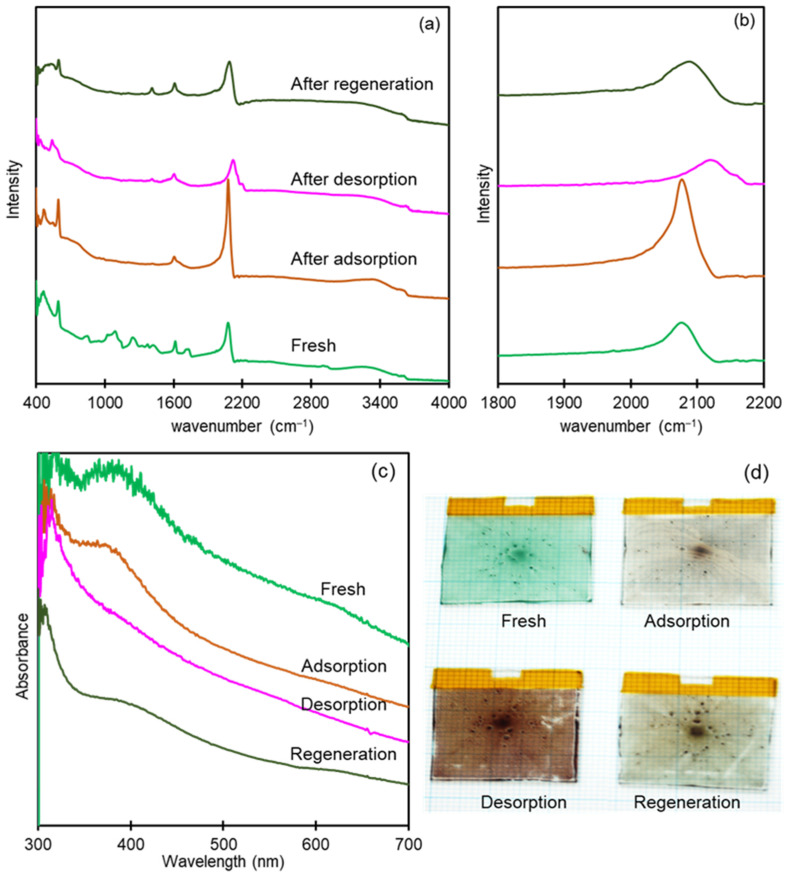
Characterization of CoHCF thin film during each step of the adsorption-desorption-regeneration cycle. (**a**,**b**) FTIR spectrum, (**c**) UV-vis spectrum, and (**d**) photographs of CoHCF thin films at fresh state, after adsorption, desorption, and regeneration.

FTIR and UV-Vis spectrum of CoHCF modified electrode before adsorption and after adsorption, desorption, and regeneration were shown in Figure 5a–c. The color change during different reaction processes was demonstrated in Figure 5d. 

According to the FTIR spectrum, the characteristic peak for CoHCF of O-H stretching (2800–4000 cm^−1^), bending (~1600 cm^−1^) and C-N stretching (2080 cm^−1^) vibration can be found [30,43]. As shown in Figure 5b, the FTIR peak for the cyanide group was seen to have a wavenumber shift and shape change after desorption and regeneration. Being an extensively reported phenomenon, we already knew that the different wavenumber of the cyanide peak corresponds to the different valence state of Fe/Co in CoHCF, and the peaks shifting from a lower to a higher wavenumber indicated the transformation of Co(II)-Fe(II) state to Co(III)-Fe(II), and then to Co(II)-Fe(III) [26]. In this study, it was clear that the cyanide peak remained unchanged at 2076 cm^−1^ before and after dipping the CoHCF thin film into seawater. After the desorption, the peak was right-shifted to 2119 cm^−1^ and 2159 cm^−1^, suggesting that by applying electrochemical potential of 1.0 V vs. Ag/AgCl, the original CoHCF thin film of Co(II)-Fe(II) was partially oxidized into Co(III)-Fe(II) and Co(II)-Fe(III) states. In addition, at the regeneration process where a reduced potential of −0.2 V vs. Ag/AgCl was applied, the cyanide group returned to 2088 cm^−1^, representing a recovery of Co(II)-Fe(II) state.

From the UV-vis spectrum, the original CoHCF thin-film demonstrated two absorbances at 380 nm and 620 nm. After being dipped into seawater, the exchange of K^+^ with Na^+^ inside the CoHCF lattice resulted in the disappearance of the 620 nm peak. The spectral difference was also reflected by the color change of CoHCF thin film, which changed from olive green to light brown. The alkaline cation influence on the UV-vis spectrum and color of CoHCF was consistent with previous findings [30,44]. After the electrochemical oxidation process, both absorbances of 380 nm and 620 nm in the UV-Vis spectrum was obviously decreased. At the same time, CoHCF thin-film transformed from light brown to purple-red, consistent with results reported before [26]. After the electrochemical regeneration of CoHCF thin film by applying low potential, the UV-Vis spectrum returned to a form similar to that of the fresh electrode. However, the intensity of 620 nm absorbance was relatively lower compared with the fresh one, while for the thin film color, it displayed a mix of the original olive-green state and the light brown state, indicating the remaining of K^+^ cations in CoHCF lattice although a part of NaCoHCF has been recovered.

Either from FTIR/UV-visible spectrum obtained, or simply from the observation of color change for the CoHCF thin film, we can draw to the conclusion that under the application of electrochemical potential, oxidation and reduction were at least partially happening on the CoHCF thin film. Moreover, according to the electrochemical switched ion-exchange property of CoHCF, the release or regain of alkaline ions (Na^+^ and K^+^ in this study) in CoHCF thin film is predicted to occur at the same time.

### 3.5. Mechanism of Electrochemical K^+^ Recovery and Film Regeneration

From the discussion in Section 3.4, we have already known that the redox reaction of CoHCF thin film is related to the inclusion/release of alkaline ions (Na^+^ and K^+^). According to the principle of charge balance, the oxidation of CoHCF will be accompanied by loss of alkaline ions in CoHCF lattice while conversely, the electrochemical reduction reaction will cause the alkaline ions in the electrolyte to go into CoHCF lattice.

Under the preparation condition at R_mix_ = 0.67, the ratio of Fe/Co in CoHCF thin film should be around 0.67, which was quite close to the results in Table 4. As indicated by the composition analysis and spectroelectrochemical measurement, the mechanism of electrochemical reaction happening on CoHCF thin film during adsorption, desorption and regeneration process was assumed to comply with Equations (4)–(8).

Adsorption:

(4) $${\mathrm{Na}}_{0.67}{\mathrm{Co}}^{\mathrm{II}}{\left[{\mathrm{Fe}}^{\mathrm{II}}{\left(\mathrm{CN}\right)}_{6}\right]}_{0.67}+0.67K^{+}\to K_{0.67}{\mathrm{Co}}^{\mathrm{II}}{\left[{\mathrm{Fe}}^{\mathrm{II}}{\left(\mathrm{CN}\right)}_{6}\right]}_{0.67}+0.67{\mathrm{Na}}^{+}$$

Desorption:

(5) $$K_{0.67}{\mathrm{Co}}^{\mathrm{II}}{\left[{\mathrm{Fe}}^{\mathrm{II}}{\left(\mathrm{CN}\right)}_{6}\right]}_{0.67}\to {\mathrm{Co}}_{0.33}^{\mathrm{II}}{\mathrm{Co}}_{0.67}^{\mathrm{III}}{\left[{\mathrm{Fe}}^{\mathrm{II}}{\left(\mathrm{CN}\right)}_{6}\right]}_{0.67}+0.67K^{+}+0.67e^{-}$$

(6) $$K_{0.67}{\mathrm{Co}}^{\mathrm{II}}{\left[{\mathrm{Fe}}^{\mathrm{II}}{\left(\mathrm{CN}\right)}_{6}\right]}_{0.67}\to {\mathrm{Co}}^{\mathrm{II}}{\left[{\mathrm{Fe}}^{\mathrm{III}}{\left(\mathrm{CN}\right)}_{6}\right]}_{0.67}+0.67K^{+}+0.67e^{-}$$

Regeneration:

(7) $${\mathrm{Co}}_{0.33}^{\mathrm{II}}{\mathrm{Co}}_{0.67}^{\mathrm{III}}{\left[{\mathrm{Fe}}^{\mathrm{II}}{\left(\mathrm{CN}\right)}_{6}\right]}_{0.67}+0.67e^{-}+0.67{\mathrm{Na}}^{+}\to {\mathrm{Na}}_{0.67}{\mathrm{Co}}^{\mathrm{II}}{\left[{\mathrm{Fe}}^{\mathrm{II}}{\left(\mathrm{CN}\right)}_{6}\right]}_{0.67}$$

(8) $${\mathrm{Co}}^{\mathrm{II}}{\left[{\mathrm{Fe}}^{\mathrm{III}}{\left(\mathrm{CN}\right)}_{6}\right]}_{0.67}+0.67e^{-}+0.67{\mathrm{Na}}^{+}\to {\mathrm{Na}}_{0.67}{\mathrm{Co}}^{\mathrm{II}}{\left[{\mathrm{Fe}}^{\mathrm{III}}{\left(\mathrm{CN}\right)}_{6}\right]}_{0.67}$$

Comparing the theoretical composition change of CoHCF with results shown in Table 4, some differences in the real case can be observed. The composition of CoHCF at a fresh state was identified to be Na_0.85_Co[Fe(CN)_6_]_0.65_ (H_2_O omitted). The higher Na content (x value) than the theoretical value from charge balance calculation was because of the remaining NaCl during the preparation process. After dipping the CoHCF thin film modified electrode in seawater, Na^+^ was almost completely exchanged by K^+^ and the molecule composition turned to a neutrally-charged one Na_0.005_K_0.68_Co[Fe(CN)_6_]_0.672_, reflecting a higher selectivity toward K^+^ compared with Na^+^ by CoHCF thin film. The composition became Na_0.003_K_0.22_Co[Fe(CN)_6_]_0.643_ accompanied by electrochemical desorption. It was obvious that the amount of K element in CoHCF was largely depleted corresponding to the oxidation of Co(II)-Fe(II) to Co(II)-Fe(III)/Co(III)-Fe(II). Still, there was around 30% of the K^+^ remained in the CoHCF lattice, explaining a part of the decrease for K^+^ adsorption ability of CoHCF with increasing recycling time (Figure 3c). Furthermore, incomplete oxidation of Co(II)-Fe(II) was reflected by the existence of positively charged ion K^+^. After the regeneration process, the composition was transformed into Na_0.411_K_0.203_Co[Fe(CN)_6_]_0.680_. A part of the Na^+^ ions was regained into the CoHCF lattice and the recovery of alkaline cations indicated the reduction of Co(II)-Fe(III)/Co(III)-Fe(II) back to Co(II)-Fe(II), which is consistent with our prediction of Equations (5) and (6).

The ratio of Fe/Co in the CoHCF composition remained almost unchanged in the four states of Table 4, reflecting the stability of the Fe-Co framework of CoHCF. The selective K^+^ adsorption ability of CoHCF from seawater was revealed by the composition analysis results. It is also proved that the electrochemical oxidation of CoHCF was accompanied by alkaline cations release, while the reduction process was with alkaline cation adsorption.

## 4. Conclusions

The study brought up a new system based on the CoHCF thin film modified electrode, which was proved to be effective in K^+^ recovery from seawater for at least five cycles. First, the excellent K^+^ adsorption ability by CoHCF was demonstrated in seawater. On the other hand, cycle adsorption experiments were carried out and the K^+^ adsorption ability first decreased fast from the first to the second cycle, and was then kept relatively stable. The K^+^ adsorption remained above 75% from the second the fifth cycle. Finally, the mechanism of K^+^ adsorption/desorption and adsorbent regeneration was elucidated. It was found that K^+^ desorption was accompanied by the oxidation of Fe(II)-Co(II), while adsorbent regeneration happened with the reduction of the material. The system is significant not only for its high selectively K^+^ adsorption ability but also for the minimization of other substances introduced into the system, making it convenient for obtaining high purity potash products. In the following research, we will focus on enlarging the area of the CoHCF thin film modified electrode and enhance the volume or amount of seawater able to be treated by the system. 

## Figures and Tables

**Table 1:** Main components of the artificial seawater used in this experiment.

Component	Concentration (ppm)	Component	Concentration (ppm)
MgCl_2_·6H_2_O	1328.41	KBr	33.02
CaCl_2_·2H_2_O	418.47	H_3_BO_3_	4.72
SrCl_2_·6H_2_O	13.97	NaF	1.64
KCl	364.23	NaCl	9651.06
NaHCO_3_	55.01	Na_2_SO_4_	662.64

**Table 2:** Particle size of CoHCF thin film estimated from peaks of XRD patterns.

Plane	Peak 2θ (deg)	FWHM (deg) *	Size (nm)	Average Size (nm)
200	17.41	0.32	249.6	-
220	24.66	0.49	165.8	178.4
420	39.55	0.70	119.9	-

* FWHM: Full width of half maximum of the peak.

**Table 3:** Faraday efficiency of electrochemical oxidation reaction during desorption process of CoHCF thin film.

Cycle Number	Q_red_/C	Q_meas_/C	FE/%
1	0.255	0.687	37.07
2	0.173	0.381	45.29
3	0.180	0.291	61.76
4	0.137	0.297	46.12
5	0.131	0.205	63.84

**Table 4:** Change of chemical composition of CoHCF thin film in the first adsorption-desorption-regeneration cycle at fresh state, after adsorption, desorption, and regeneration, the formula of CoHCF denoted as Na_x_K_y_Co[Fe(CN)_6_]_z_.

State of the CoHCF Thin Film	x (Na)	y (K)	z (Fe)
Fresh	0.850	0	0.650
After adsorption	0.005	0.680	0.672
After desorption	0.003	0.220	0.643
After regeneration	0.411	0.203	0.680

## Data Availability

Data sharing is not applicable for this article.
